# Discovery of BAR502, as potent steroidal antagonist of leukemia inhibitory factor receptor for the treatment of pancreatic adenocarcinoma

**DOI:** 10.3389/fonc.2023.1140730

**Published:** 2023-03-14

**Authors:** Cristina Di Giorgio, Rachele Bellini, Antonio Lupia, Carmen Massa, Martina Bordoni, Silvia Marchianò, Rosalinda Rosselli, Valentina Sepe, Pasquale Rapacciuolo, Federica Moraca, Elva Morretta, Patrizia Ricci, Ginevra Urbani, Maria Chiara Monti, Michele Biagioli, Eleonora Distrutti, Bruno Catalanotti, Angela Zampella, Stefano Fiorucci

**Affiliations:** ^1^ Department of Medicine and Surgery, University of Perugia, Perugia, Italy; ^2^ Department of Pharmacy, University of Naples Federico II, Naples, Italy; ^3^ Net4Science srl, University “Magna Græcia”, Catanzaro, Italy; ^4^ Department of Pharmacy, University of Salerno, Salerno, Italy; ^5^ Department of Gastroenterology, Azienda Ospedaliera di Perugia, Perugia, Italy

**Keywords:** PDAC, LIF, LIFR, bile acids, steroid, proliferation, EMT

## Abstract

**Introduction:**

The leukemia inhibitory factor (LIF), is a cytokine belonging to IL-6 family, whose overexpression correlate with poor prognosis in cancer patients, including pancreatic ductal adenocarcinoma (PDAC). LIF signaling is mediate by its binding to the heterodimeric LIF receptor (LIFR) complex formed by the LIFR receptor and Gp130, leading to JAK1/STAT3 activation. Bile acids are steroid that modulates the expression/activity of membrane and nuclear receptors, including the Farnesoid-X-Receptor (FXR) and G Protein Bile Acid Activated Receptor (GPBAR1).

**Methods:**

Herein we have investigated whether ligands to FXR and GPBAR1 modulate LIF/LIFR pathway in PDAC cells and whether these receptors are expressed in human neoplastic tissues.

**Results:**

The transcriptome analysis of a cohort of PDCA patients revealed that expression of LIF and LIFR is increased in the neoplastic tissue in comparison to paired non-neoplastic tissues. By *in vitro* assay we found that both primary and secondary bile acids exert a weak antagonistic effect on LIF/LIFR signaling. In contrast, BAR502 a non-bile acid steroidal dual FXR and GPBAR1 ligand, potently inhibits binding of LIF to LIFR with an IC_50_ of 3.8 µM.

**Discussion:**

BAR502 reverses the pattern LIF-induced in a FXR and GPBAR1 independent manner, suggesting a potential role for BAR502 in the treatment of LIFR overexpressing-PDAC.

## Introduction

Pancreatic ductal adenocarcinoma (PDAC) represents the ≈ 85% of pancreatic cancer (PC) but is projected to become the second leading cause of cancer death in industrialized countries by 2030 (1, 2). Due to a late diagnosis (3), ≈90% of PDAC are detected at an advanced stage beyond the criteria for curative surgery (4). PDAC risk factors include high alcohol consumption, smoking, a sedentary life style and chronic high caloric intake, obesity, diabetes, hypertriglyceridemia, biliary stones and acute recurrent and chronic pancreatitis (5). The PDAC has also a strong genetic background and associates with several somatic mutations in oncogenes and tumour suppressor genes, including: KRAS, TP53, CDKN2A/p16, and SMAD4 (6). Most commonly, PDAC patients develop resistance to chemotherapy, making the identification of mechanistic molecular pathways and putative biomarkers an urgent need (7).

Next generation sequencing studies of PDAC have identified several markers linked to patient’s survival. Transcriptome studies have identified the Leukaemia Inhibitory Factor (LIF) as a potential biomarker of poor prognosis in PDAC patients. LIF is a pleiotropic member of interleukin (IL)-6 cytokine family (8), that regulates cell differentiation, proliferation and survival in embryo and adult cells and is involved in cancer growth and progression (9). LIF signalling is mediated *via* binding to an heterodimeric LIF receptor (LIFR) complex, formed by LIFR and the glycoprotein (gp) 130. This complex is also targeted by other potential oncogenic factors including oncostatin M, cardiotropin 1 (CT1) and neutrophil ciliary factor (CNTF) and the cardiotropin-like cytokine factor (CLCF1) whose expression and activity has been detected in several tumors (10). Upon binding to its ligands, LIFR undergoes a series of conformational rearrangements that promote the phosphorylation of the Jak-Tyk, two proteins that are constitutively associated to cytoplasmic domain of the gp130/LIFR complex (11), activating the downstream signalling pathways which include JAK1/STAT3, MAPK and AKT. The LIF/LIFR axis and JAK/STAT3 signalling pathway is over-regulated in several type of solid tumours, including PDAC (12), gastric cancer (GC) (13), hepatocellular carcinoma (HCC) (14), colon-rectal cancer (CRC) (15) and breast cancer (16), and promotes cancer cell proliferation, epithelial-to-mesenchymal transition (EMT) (17) and regulates aberrantly the self-renewal of cancer cell-initiating tumors (18), as well as promoting radio (19) and chemo-resistance (15). Several studies support the suppression of LIFR signalling as potential target in inhibiting cell growth and tumour progression (9), and we have shown recently, that LIFR-mediated antagonism supports the anti-oncogenic effect of mifepristone in pancreatic cancer and chemoresistance (20). Furthermore, while LIFR antagonists are not approved for clinical use (21), several anti-LIFR molecules are investigated in phase II and III clinical trials in various oncologic settings (22).

Bile acids are steroid derivatives of cholesterol, synthetized in the liver and metabolized by microbiota hydrolase in the intestine (e.g from Bacteroidetes, Clostridium and Enterococcus) (23) and reabsorbed through the enterohepatic circulation (24). Despite, secondary bile acids (lithocholic and cholic acid, LCA and DCA) are traditionally considered as potential causative factors for development of gastrointestinal cancers (25), more recent studies have reported that bile acids exert robust anti-tumor effects (26). The effects that various bile acid species exert on cancer growth and progression are dependent on their concentrations and cellular environment as well as differential expression of their main receptors, the Farnesoid-X-Receptor (FXR) (27–29) and the bile acid activated G protein coupled receptor (GPBAR1) (30, 31). Generally, while high concentrations of bile acids promote cells injury and cell proliferation, lower concentrations, corresponding to their plasmatic or tissue concentrations, exert anticancer activity in a large subset of gastrointestinal malignancies (32). It has been previously reported that UDCA (0.25-1 mM) promotes apoptosis of gastric cancer cell lines such as SNU601 and SNU638 cells (33) and MKN45 cells, while DCA (200 μM) induces MUC2 expression and inhibits tumour invasion and migration in colon cancer cells (CRC) (34). In several *in vitro* models of CRC, UDCA (0.2 mM) (35) and DCA (36) induce apoptosis and inhibit cell proliferation. In the same manner, TUDCA (50 μg/ml) suppresses NF-kB signalling and ameliorates colitis-associated tumorigenesis (37) and LCA (150-400 μM) (38), DCA (500 μM) and CDCA (500 μM) inhibit cell growth and induce programmed cell death (39). It is worth noting that UDCA reduced intracellular ROS levels and Prx2 expression, as well as suppresses EMT process and interferes with “self-renewal” ability of cancer stem cells (CSC), in pancreatic cancer cell lines such as HPAC and Capan1 cells (40).

Building on the background that steroidal-like agents such as mifepristone and EC359 exert LIFR antagonist effects, we have evaluated the molecular docking of an in-house library based on both natural and synthetic bile acids on hLIFR, and found that LCA and CDCA act as weak LIFR antagonists. Additionally, we have shown BAR502 (41, 42), a semisynthetic bile alcohol steroidal agonist of FXR and GPBAR1, as a potential hLIFR antagonist acting as a tumour suppressor and reverting proliferation and EMT process in a LIFR-dependent manner.

## Materials and methods

### GSE196009 data sets

The GSE196009 repository (https://www.ncbi.nlm.nih.gov/geo/query/acc.cgi?acc=GSE196009) accessed on 1 August 2022 includes gene expression profiles (RNA-seq analysis, Illumina HiSeq 2000) of fresh or frozen PDAC tissues and adjacent normal pancreatic tissues from 12 Japanese patients.

### Alpha screen

Recombinant human LIFR (His Tag) and biotinylated recombinant human LIF were purchased from Sino Biologicals (Sino Biological Europe GmbH, Dusseldorf, Germany) and R&D Systems (Abingdon, UK), respectively, and both were reconstituted as required by the manufacturer. Inhibition of LIFR/LIF binding by LCA, CDCA, PDL103 and BAR502 was measured by Alpha Screen (Amplified Luminescent Proximity Homogeneous Assay). The assay was carried out in white, low-volume, 384-well AlphaPlates (PerkinElmer, Waltham, MA, USA) using a final volume of 25 µL and an assay buffer containing 25 mM Hepes (pH 7.4), 100 mM NaCl, and 0.005% Kathon. The concentration of DMSO in each well was maintained at 5%. LIFR (His Tag, final concentration 4.5 nM) was incubated with LCA, CDCA, PDL103, a dual FXR/GPBAR1 antagonist, and BAR502 or a vehicle for 45 min under continuous shaking. Then, LIF was added (biotinylated, final concentration 9 nM), and the samples were incubated for 15 min prior to adding His-Tag acceptor beads (final concentration 20 ng/µL) for 30 min. Then, streptavidin donor beads were added (final concentration 20 ng/µL), and the plate was incubated in the dark for 2 h and then read in an EnSpire Alpha multimode plate reader (PerkinElmer, Waltham, MA, USA).

### Transactivation assay

To perform STAT3 transactivation, HepG2 (HB, 8065 from ATCC), an immortalized human epatocarcinoma cell line was used, as described previously (20). On day 0, HepG2 were seeded at 7.5 × 104 cells/well in a 24-well plate and maintained at 37°C and 5% CO2 in E-MEM supplemented with 10% FBS, 1% glutamine, and 1% penicillin/streptomycin. On day 1, cells were transiently transfected with the reporter plasmid pGL4.47[luc2P/SIE/Hygro] (200 ng) (CAT#: E4041 Promega, Madison, WI, USA), a vector encoding the hLIFR (CAT# RC226327) (100 ng) and CD130 (IL6ST) (100 ng) (CAT#: RC215123, OriGene Technologies, Inc. Rockville, MD, USA), and finally a vector encoding the human RENILLA luciferase gene (pGL4.70) (100 ng) (Promega, Madison, WI, USA). On day 2, cells were exposed to the cytokine LIF (10 ng/mL) alone or in combination with BAR502 (from 0.1 to 20 μM). To investigate the GPBAR1 activation, HEK-293T cells were transiently transfected with 200 ng of human pGL4.29 (Promega), a reporter vector containing a cAMP response element (CRE) that drives the transcription of the luciferase reporter gene luc2P, with 100 ng of pCMVSPORT6-human GPBAR1 and with 100 ng of pGL4.70 as described previously (42, 43). For FXR mediated transactivation, HepG2 cells were plated at 7.5 × 104 cells/well in a 24 well plate. Cells were transfected with 200 ng of the reporter vector p(hsp27)-TK-LUC containing a FXR response element (IR1) cloned from the promoter of heat shock protein 27 (hsp27), 100 ng of pSG5-FXR, 100 ng of pSG5-RXR, and 100 ng of pGL4.70 (Promega), a vector encoding the human Renilla gene. To perform STAT3 transactivation, HepG2 were seeded at 7.5× 104 cells/well in a 24-well plate. On the day-1, cells were transiently transfected with 200 ng of the reporter plasmid pGL4.47[luc2P/SIE/Hygro] (CAT#: E4041 Promega, Madison, WI, USA), 100 ng of a vector encoding the hLIFR (CAT# RC226327) and 100 ng of CD130 (IL6ST) (CAT#: RC215123, OriGene Technologies, Inc. Rockville, MD USA), and finally with 100 ng of a vector encoding the human RENILLA luciferase gene (pGL4.70) (Promega, Madison, WI, USA). At 24 h post-transfection, HepG2 and HEK293T were stimulated 18 h with Taurolithocholic Acid (TLCA,10 μM) or Chenodeoxycholic acid (CDCA, 10 μM) or Leukemia Inhibitory factor (LIF, 10 ng/ml) as positive controls and compound PDL103 at increasing concentrations (from 0.1 μM to 100 μM) in combination with the relative positive controls. Then, after 24 h, the cells were lysed in 100 μL of lysis buffer (25 mM Tris-phosphate, pH 7.8; 2 mM dithiothreitol (DTT); 10% glycerol; 1% Triton X-100). Then, 10 μL cellular lysates were assayed for luciferase and RENILLA activities using the Dual-Luciferase Reporter assay system (Promega, Madison, WI, USA). Luminescence was measured using a Glomax 20/20 luminometer (Promega, Madison, WI, USA). LUCIFERASE activities (RLU) were normalized with RENILLA activities (RRU).

### Computational studies

#### Protein and ligand preparations

The three-dimensional (3D) crystallographic structures of the human LIFR, hLIFR (Uniprot ID Code: P42702, PDB X-Ray 3E0G [REF DOI: 10.1186/1756-9966-28-83) was retrieved from the RCSB Protein Data Bank (www.rcsb.org). The downloaded structure was subjected to Maestro’s *Protein Preparation Wizard* (PPW) tool (Schrödinger Release 2021-1) to assign bond orders, add hydrogen atoms, adjust disulphide bonds, add caps to chains break, and assign residues protonation state at pH 7.4. The in-house library of natural and synthetic bile acids (Bile acids) was prepared using LigPrep (LigPrep. Schrödinger, release 2021–1, LigPrep; Schrödinger, LLC: New York, NY, USA, 2021) and Epik (Schrödinger; Release 2021-1: Epik, S., LLC, New York, NY, USA, 2021) modules to generate and optimize the 3D structures of the ligands at the protonation states of pH 7.4.

#### Docking procedures

The optimized structure of hLIFR was used for the accurate QM-Polarized Ligands Docking (QPLD) (Glide, S., LLC, New York, NY, USA, 2021; Jaguar, S., LLC, New York, NY, USA, 2021) and Induced Fit Docking (IFD) (Glide, S., LLC, New York, NY, USA, 2021; Prime, S., LLC, New York, NY, USA, 2021) docking protocols, following the same procedures described in our previous work (Di Giorgio et al.). Briefly, the centroid of the hLIFR binding site was used to generate the grid box coordinate in default size (10.0 Å). Ten docking poses were saved for each ligand of the in-house library after the QPLD process, and the most representatives were submitted to the IFD procedure using the extended sampling protocol. A maximum of 80 poses was generated, and the energy window for the ligand conformational sampling was 2.5 kcal/mol.

### Molecular dynamics simulations

The best scored IFD docking pose of BAR502 was subjected to 100 ns of MDs. The CUDA version of the AMBER18 package (44) was used to MD simulation, using the Amber ff14SB force field (45, 46) to treat the protein. Ligand charges were, instead, calculated using the restrained electrostatic potential (RESP) fitting procedure (40). The Gaussian16 package (47): was used to calculate the ligand ESP using the 6-31G* bile acids is set at the Hartree-Fock level of theory. Antechamber (48): coupled with the general amber force field (GAFF2) parameters (49), allowed RESP charges and the ligand force field parameters. The system was solvated in a 10 Å layer of the octahedral box using TIP3P (50): water molecules parameters. The SHAKE algorithm was used to constraint bonds involving hydrogen atoms with two fs integration time steps. Next, the system was minimized and thermally equilibrated as described in our latest work (20). The MD trajectory was visualized by using Visual Molecular Dynamics (VMD) graphics ver. 1.9.3 (51), while clustering and analysis procedures were performed through the CPPTRAJ module (52). For the most representative cluster population, intermolecular interaction energy was analysed *via* the Molecular Mechanics/Generalized Born Surface Area (MM/GBSA) equation (53). All images were rendered using Maestro GUI Suite 2021-1 (Schrödinger Release 2021-1) and Adobe Illustrator (Adobe Systems, San Jose, CA, USA).

### Cell lines

Human pancreatic cell lines MIA-PaCa-2 and PANC-1 were from ATCC (Manassas, VA; USA). The cells were grown in DMEM (Sigma-Merk LIFe Science S.r.l. Milan, Italy) medium supplemented with 10% Fetal Bovine Serum (FBS), 1% L-Glutamine, 1% Penicillin/Streptomycin, in a humidified 5% CO_2_ atmosphere, 37°C. U-937 a cell line exhibiting monocyte morphology were purchased from Sigma Aldrich (Sigma-Merk LIFe Science S.r.l. Milan, Italy). U937 and MKN45 were grown in RPMI complete medium, supplemented with 10% FBS, 1% L-Glutamine, 1% Penicillin/Streptomycin. A human hepatocarcinoma cell line, HEPG2 (ATCC) was grown at 37°C in E-MEM complete medium containing 10% FBS, 1% L-glutamine and 1% penicillin/streptomycin. Cells are free from Mycoplasma contamination as confirmed by Mycoplasma PCR Detection test (Sigma-Merk LIFe Science S.r.l. Milan, Italy) and were regularly passaged to maintain exponential growth and used from early passages (<10 passages after thawing). In all experiments, cells were serum starved for 24 h before exposure to tested agent.

### Real-time PCR

The RNA was extracted from cell lines using and Direct-zol™ RNA MiniPrep w/Zymo-Spin™ IIC Columns (Zymo Research, Irvine, CA, USA)., according to the manufacturer’s protocol as described previously (20). After purification from genomic DNA by DNase-I treatment (ThermoFisher Scientific, Waltham, MA USA), 2 µg of RNA from each sample was reverse-transcribed using Kit FastGene Scriptase Basic (Nippon Genetics, Mariaweilerstraße, Düren, Germania) in a 20 μL reaction volume. Finally, 50 ng cDNA were amp LIFied in a 20 μL solution containing 200 nM of each primer and 10 μL of SYBR Select Master Mix (ThermoFisher Scientific). All reactions were performed in triplicate, and the thermal cycling conditions were as follows: 3 min at 95°C, followed by 40 cycles of 95°C for 15 s, 56°C for 20 s and 72°C for 30 s, using a Step One Plus machine (Applied Biosystem). The relative mRNA expression was calculated accordingly to the 2^-ΔCt^ method. Primers used in this study were designed using the PRIMER3 (http://frodo.wi.mit.edu/primer3/) software using the NCBI databile acids e. RT-PCR primers used in this study for human sample and human cell lines were as follow [forward (for) and reverse (rev)]:

LIFR (for GCTCGTAAAATTAGTGACCCACA; rev GCACATTCCAAGGGCATATC),

LIF (for CCCTGTCGCTCTCTAAGCAC; rev GGGATGGACAGATGGACAAC),

GPBAR1 (for ACTGCAGCTCCCAGGCTAT; rev GACAGAGAGGAAGGCAGCA),

FXR (for GCAGCCTGAAGAGTGGTACTCTC; rev CATTCAGCCAACATTCCCATCTC),

SNAIL1 (for ACCCACACTGGCGAGAAG; rev TGACATCTGAGTGGGTCTGG),

VIMENTIN (for TCAGAGAGAGGAAGCCGAAA; rev ATTCCACTTTGCGTTCAAGG),

CXCR4 (for AACGTCAGTGAGGCAGATGA; revTGGAGTGTGACAGCTTGGAG).

### Immunofluorescence

Immunofluorescence (IF) staining was carried out using MIA PaCa-2 cells. Cells cytospins were fixed in methanol for 20 min and then washed 3 times with phosphate buffered saline (PBS 1X), permeabilized and then incubated with Blocking buffer (PBS 1X with 10% horse serum and 1% BSA) for 1h at room temperature. Primary antibodies, anti- GPBAR1 (NBP2-23669), (Novus Biologicals) and anti-FXR (ORB156973) (Biorbyt) were dissolved in Blocking Buffer and incubated overnight at 4°. On the following day cells were washed three times with PBS 1X containing 0,1% Tween 20 (PBST), and then incubated with the secondary antibody, Goat anti-rabbit IgG (H + L) Alexa Fluor 488 (ab150077) (Abcam) for GPBAR1 and Goat anti-rabbit IgG (H + L) Alexa Fluor 568 (A11011) for FXR (Invitrogen), diluted in Blocking Buffer for 1h at room temperature in the dark. After 3 washes with PBST, nucleus was counterstained with DAPI 1X for 1 min in the dark and the reaction was stopped by a final wash in PBS 1X for 5 min. Then, slides were mounted with ProLong Glass Antifade Mountant (P36980) (Invitrogen, Thermofisher scientific Waltham, Massachusetts, USA), sealed with nail polish and observed at fluorescence microscope (Olympus BX60, Rome, Italy).

### Cell proliferation assay

The cell viability assay was done using the CellTiter 96 Aqueous One Solution Cell Proliferation Assay (Promega, Milano, Italy), a colorimetric method for accessing the number of viable cells in proliferation as described previously (13). MIA-PaCa 2 cells were seeded in DMEM complete medium at 36 *10^3^ cells/100 uL well into 96-well tissue culture plate. After 24 h, cells were serum starved for 24 h and then were primed with the LIFR major ligand, LIF (10 ng/ml) alone or in combination with BAR502 (5,10 and 20 μM) or only with vehicle. In another experimental setting, MIA-PaCa 2 cells were triggered with PDL103 (10 μM) alone, LIF (10 ng/ml) alone or plus PDL103 or BAR502 (10 μM) or both. In a different setting cell were exposed to an antagonist of the Farnesoid X receptor (FXR), 3-(naphthalen-2-yl)-5-(piperidin-4-yl)-1,2,4-oxadiazole (GP7) (10 µM) (54), LIF (10 ng/ml) alone or in combination with GP7 or BAR502 or both. Then cell proliferation assessed as mentioned above. Absorbance was measured using a 96 well reader spectrophotometer (490 nm). In these experiments each experimental setting was replicated ten folds. For analysis the background readings with the medium alone, were subtracted from the samples read-outs.

### Flow cytometry

MIA-PaCa2 cells were seeded in 6-well tissue culture plate (cell density 700 × 103/well) and cultured as specified above. Cells were serum-starved for 8 h and then incubated with LIF (10 ng/mL) alone or plus BAR502 (10, 20 µM) or a vehicle for 24 h. The intracellular flow cytometry staining for Ki-67 was performed using the following reagents: Ki-67 Monoclonal Antibody (SolA15), Alexa Fluor™ 488, (eBioscience™, San Diego, California, USA) and 7-AAD to characterize the cell cycle phases G0-G1 and S-G2-M. Before intracellular IC-FACS, staining cells were fixed for 30 min in the dark using IC Fixation buffer (eBioscience™) and then permeabilized using Permeabilization buffer (10X) (eBioscience™). The staining for Annexin V was performed using the Annexin V Antibody (A13199, Thermofisher Scientific, Waltham, MA, USA) to evaluate the apoptosis rate. Briefly, 5 μL of Annexin V Antibody was added to each 100 μL of cell suspension, and cells were incubated the at room temperature for 15 min. Flow cytometry analyses were carried out using a 3-laser standard configuration ATTUNE NxT (LIFe Technologies, Carlsbad, CA, USA). Data were analyzed using FlowJo software (TreeStar) and the gates set using a fluorescence minus-one (FMO) control strategy. FMO controls are samples that include all conjugated Abs present in the test samples except for one. The channel in which the conjugated Ab is missing is the one for which the fluorescence minus one provides a gating control.

### Western blot analysis

MIA-PaCa 2 cells were seeded in 6-well tissue culture plate (cell density 1.5 * 10^6^/well) in DMEM complete medium. After serum starving, cells were incubated with LIF (10 ng/mL) alone or plus BAR502 (10 µM) for 10 min. Total lysates were prepared by homogenization of MIA-PaCa2 cells in RIPA buffer containing phosphatase and protease inhibitors. Protein extracts were electrophoresed on 12% acrylamide Tris-Glycine gel (Invitrogen), blotted to nitrocellulose membrane, and then incubated overnight with primary Abs against GAPDH (bs2188R 1:1000; Bioss antibodies), STAT3 (sc-8019 1:500; Santa Cruz Biotechnology), Vimentin (ab92547 1:1000;Abcam), phosho-Stat3 (GTX118000 1:1000; Genetex). Primary Abs were detected with the HRP-labeled secondary Abs. Proteins were visualized by Immobilon Western Chemiluminescent Reagent (MilliporeSigma) and iBright Imaging Systems (Invitrogen). Quantitative densitometry analysis was performed using ImageJ software. The degree of STAT3 phosphorylation was calculated as the ratio between the densitometry readings of Vimentin/GAPDH and p-STAT3/STAT3.

### Wound healing assay

MIA PaCa-2 cells were seeded in DMEM complete medium at 800x10^3^ cells/well into 24-well plate and used at 70-80% confluence rate. The assay was performed as previously described (20), particularly on the day 1, the cell monolayers were gently scraped vertically with a new 0.2 mL pipette tip across the centre of the well. After scratching, the well was gently washed twice with PBS (Euroclone, Milan, Italy) to remove the detached cells and cell debris and finally fresh medium containing LIF (10 ng/mL) alone or in combination with BAR502 (10 µM) or EC359 (25 nM) was added into each well. Immediately after scratch creation, the 24-plate was placed under a phase-contrast microscope and the first image of the scratch acquired (T0) with using a OPTIKAM Pro Cool 5 – 4083.CL5 camera. Cells were grown for additional 48 h and images taken at 24h (T1) and 48 h (T2). The gap distance between scarps borders was quantified by assessing that area between the two margins of the scratches. All experiments were performed in triplicate.

### Chemistry

(E)-2,6-dichlorobenzaldehyde oxime (2). A solution of hydroxylamine hydrochloride (1.5 eq) and NaOH (1.5 eq) in water was added to a solution of 2,6-dichlorobenzaldehyde (1) in ethanol. The mixture was left to stir for 5h. After starting material consumption, ethanol was evaporated, and the residue was extracted with ethyl acetate (x 3). The reunited organics were washed with brine, dried over anhydrous Na_2_SO_4_, and concentrated to afford the oxime as a white solid (98%) which was used for the next step without further purification. ^1^H NMR (400 MHz, CDCl_3_) δ 8.37 (s, 1H), 7.45 (d, *J* = 0.6 Hz, 2H), 7.28 (dd, *J* = 8.8, 7.8 Hz, 1H); ^13^C NMR (100 MHz, CDCl_3_) δ 144.24, 132.82, 130.12, 129.99, 128.32; HRMS (ESI) m/z [M+H^+^] calcd for C_7_H_5_Cl_2_NO 189.9748, found 189.9744.

(Z)-2,6-dichloro-N-hydroxybenzimidoyl chloride (3). To a solution of compound 2 in dry DMF, N-chlorosuccinimide (1.2 eq) was slowly added at 0°C. The mixture was stirred overnight and partitioned with distilled water and diethyl ether. The organic phase was dried over anhydrous Na_2_SO_4_ and concentrated to afford the chloro oxime (95%) as a colourless oil, used for the next step without purification. ^1^H NMR (400 MHz, CDCl_3_) δ 7.44 – 7.32 (m, 3H). ^13^C NMR (100 MHz, CDCl_3_) δ 143.92, 132.02, 131.73, 130.49, 128.27; HRMS (ESI) m/z [M+H^+^] calcd for C_7_H_4_Cl_3_NO 223.9358, found 223.9352.

(3-(2,6-dichlorophenyl)isoxazol-5-yl)methanol (4). To a solution of compound 3 in t-BuOH/H_2_O 1:1 were added in sequence propargylic alcohol (3 eq), CuSO_4_·5H_2_O (0.02 eq), sodium ascorbate (0.1 eq) and NaHCO­­_3_ (4 eq). The mixture’s appearance rapidly shifted from clear to opaque yellow upon the addition of the bile acids e. After 3h, the reaction was quenched by adding sat. NH_4_Cl solution and then extracted with ethyl acetate (x3). The reunited organics were washed with brine, dried over anhydrous Na_2_SO_4_, and concentrated to afford the isoxazole as a colourless oil (quantitative yield) which was used for the next step without further purification. ^1^H NMR (400 MHz, CDCl_3_) δ 7.52 (dd, *J* = 8.0, 0.7 Hz, 2H), 7.41 (dd, *J* = 8.8, 7.3 Hz, 1H), 6.61 (s, 1H), 4.79 (s, 2H). ^13^C NMR (100 MHz, CDCl_3_) δ 169.35, 158.33, 133.88, 130.31, 128.38, 128.07, 100.54, 57.56; HRMS (ESI) m/z [M+H^+^] calcd for C_10_H_7_Cl_2_NO_2_ 243.9854, found 243.9850.

(3-(2,6-dichlorophenyl)isoxazol-5-yl)methyl methanesulfonate (5). To a solution of compound 4 in dry THF were added triethylamine (4 eq) and mesyl chloride (3 eq) at -20°C. The reaction was stirred for 2h and then quenched by adding 1M HCl solution. The mixture was extracted with ethyl acetate (x3). The reunited organics were washed with brine, dried over anhydrous Na_2_SO_4_, and concentrated to afford the mesylate as an off-white solid (92%). ^1^H NMR (400 MHz, CDCl_3_) δ 7.52 (dd, *J* = 8.1, 0.7 Hz, 2H), 7.41 (dd, *J* = 8.8, 7.3 Hz, 1H), 6.59 (s, 1H), 5.52 (s, 2H), 3.15 (s, 3H). ^13^C NMR (100 MHz, CDCl_3_) δ 165.48, 158.18, 133.43, 130.31, 128.38, 127.81, 100.75, 61.12, 37.62; HRMS (ESI) m/z [M+Na^+^] calcd for C_11_H_9_Cl_2_NO_4_S 343.9527, found 343.9523.

Methyl 4’-((3-(2,6-dichlorophenyl)isoxazol-5-yl)methoxy)-[1,1’-biphenyl]-4-carboxylate (6). Compound 5 was dissolved in dry DMF and methyl 4’-hydroxy-4-biphenylcarboxylate (1.2 eq) and K_2_CO_3_ (2 eq) were added. The reaction was stirred at 100°C for 8h, then distilled water was added and the mixture was extracted with ethyl acetate (x3). The reunited organics were washed with brine, dried over anhydrous Na_2_SO_4_, and concentrated. The crude product was purified by flash column chromatography (silica gel, ethyl acetate/petroleum ether 15:85) to yield compound 1 (66%) as a white solid. An analytical sample was analysed by HPLC purification on a Nucleodur 100-5 column (5 μm; 10 mm i.d. x 250 mm) eluting with n-hexane/ethyl acetate 85:15 v/v (flow rate 3 mL/min, t_R_ = 19.5 min). ^1^H NMR (400 MHz, CDCl_3_) δ 8.09 (m, 2H), 7.61 (m, 4H), 7.42 (dd, *J* = 8.0, 1.0 Hz, 2H), 7.34 (dd, *J* = 9.0, 7.1 Hz, 1H), 7.10 (m, 2H), 6.48 (s, 1H), 5.31 (s, 2H), 3.94 (s, 3H). ^13^C NMR (100 MHz, CDCl_3_) δ 168.20, 167.16, 159.09, 158.13, 145.03, 135.69 (x2), 133.90, 131.32, 130.29 (x2), 128.72 (x2), 128.68, 128.41 (x2), 128.24, 126.75 (x2), 115.52 (x2), 105.18, 61.83, 52.26; HRMS (ESI) m/z [M+H^+^] calcd for C_24_H_17_Cl_2_NO_4_ 454.0535, found 454.0531.

(4’-((3-(2,6-dichlorophenyl)isoxazol-5-yl)methoxy)-[1,1’-biphenyl]-4-yl)methanol (PDL103). To a solution of compound 6 in dry THF were added dry MeOH (3 eq) and 1M LiBH_4_ (3 eq) in THF at 0°C. The reaction was left stirring overnight. The mixture was quenched by adding 1M NaOH solution (3 eq) at 0°C and then was extracted with ethyl acetate (x 3). The reunited organics were washed with brine, dried over anhydrous Na_2_SO_4_, and concentrated to afford PDL103 (82%). An analytic sample was obtained by HPLC on a Phenomenex Luna C18 (5 μm; 250 mm x 4.6 mm) column in gradient (t_0 min_= 60% B – t_3 min_= 60% B – t_25 min_= 95% B – t_30 min_= 95% B, solvent B = MeOH + 0.1% TFA, flow rate 1 mL/min, t_R_ = 22 min). ^1^H NMR (400 MHz, CDCl_3_) δ 7.56 (m, 4H), 7.43 (m, 4H), 7.33 (dd, *J* = 9.0, 7.1 Hz, 1H), 7.07 (d, *J* = 8.8 Hz, 2H), 6.47 (s, 1H), 5.30 (s, 2H), 4.74 (s, 2H). ^13^C NMR (100 MHz, CDCl3) δ 168.38, 159.08, 157.54, 140.11, 139.66, 135.69 (x2), 134.83, 131.30, 128.45 (x2), 128.40 (x2), 128.27, 127.67 (x2), 127.13 (x2), 115.44 (x2), 105.12, 65.29, 61.89; HRMS (ESI) m/z [M+H^+^] calcd for C_23_H_17_Cl_2_NO_3_ 426.0585, found 426.0580.

### AmpliSeq transcriptome

MIA PaCa-2 cells were cultured in 6-well tissue culture plate (cell density 1.5 * 10^6^/well) in DMEM complete medium. After serum starving, cells were exposed with LIF (10 ng/mL) alone or plus BAR502 (10 µM) or left untreated for 24h. High-quality RNA was extracted from MIA PaCa-2 cells using the PureLink™ RNA Mini Kit (Thermo Fisher Scientific), according to the manufacturer’s instructions. RNA quality and quantity were assessed with the Qubit^®^ RNA HS Assay Kit and a Qubit 3.0 fluorometer followed by agarose gel electrophoresis. Libraries were generated using the Ion AmpliSeq™ Transcriptome Human Gene Expression Core Panel and Chef-Ready Kit (Thermo Fisher Scientific), according to the manufacturer’s instructions. Briefly, 10 ng of RNA was reverse transcribed with SuperScript™ Vilo™ cDNA Synthesis Kit (Thermo Fisher Scientific, Waltham, MA) before library preparation on the Ion Chef™ instrument (Thermo Fisher Scientific, Waltham, MA). The resulting cDNA was amplified to prepare barcoded libraries using the Ion Code™ PCR Plate, and the Ion AmpliSeq™ Transcriptome Human Gene Expression Core Panel (Thermo Fisher Scientific, Waltham, MA), Chef-Ready Kit, according to the manufacturer’s instructions. Barcoded libraries were combined to a final concentration of 100 pM, and used to prepare Template-Positive Ion Sphere™ (Thermo Fisher Scientific, Waltham, MA) Particles to load on Ion 540™ Chips, using the Ion 540™ Kit-Chef (Thermo Fisher Scientific, Waltham, MA). Sequencing was performed on an Ion S5™ Sequencer with Torrent Suite™ Software v6 (Thermo Fisher Scientific). The analyses were performed with a range of fold <−2 and >+2 and a p value < 0.05, using Transcriptome Analysis Console Software (version 4.0.2), certified for AmpliSeq analysis (Thermo-Fisher). The transcriptomic data have been deposited as dataset on Mendeley data repository (ab92547 1:1000;Abcam).

### Statistical analysis

Statistical analysis was carried out using the one-tailed unpaired Student’s t test comparisons (* p < 0.05) using the Prism 8.0 software (GraphPad San Diego, CA, USA).

## Results

### LIF/LIFR and bile acid receptor expression in PDAC

**Figure 1:**
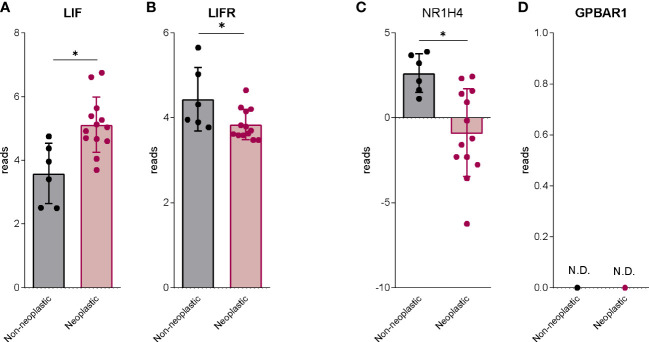
The bile acids receptors expression is downregulated in human PDAC. RNA-seq analysis of non-neoplastic and neoplastic mucosa of PDAC from GSE196009 repository. Each dot represents a patient. Data shown represent the gene profile expression of **(A)** LIF, **(B)** LIFR, Nuclear Receptor Subfamily 1 Group H Member 4 **(C)** NR1H4, The G protein-coupled bile acid receptor 1 **(D)** GPBAR1. Results are the mean ± SEM of 6 (Non-neoplastic) and 13 (Neoplastic) samples per group. *p < 0.05.

We have first investigated the expression of LIF and LIFR and the expression of FXR (NR1H4) and GPBAR1 in human PDAC. For this purpose, we have used a human repository of PDAC tissues, that includes cancer tissues along with the adjacent normal tissue excided from 12 Japanese patients (Repository GSE196009 series) (**Figure 1**). As described previously (20), LIF and LIFR show an opposite modulation in the cancer tissues, thus while LIF expression is higher in PDAC in comparison with the adjacent normal tissue (**Figure 1A**), the expression of LIFR is subject to opposite modulation (**Figure 1B**). Similarly, the expression of FXR (NR1H4) was downregulated in cancer tissues compared to the non-neoplastic tissues (**Figure 1C**). Instead, GPBAR1 was not detectable in both cancer and adjacent normal tissues (**Figure 1D**).

**Figure 2:**
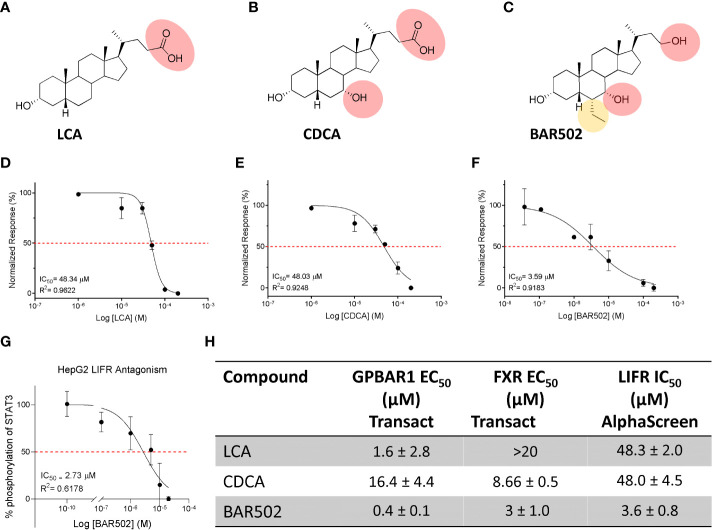
Natural and synthetic bile acids antagonize LIFR. The figure shows two-dimensional structure of **(A)** LCA **(B)** CDCA **(C)** BAR502. Natural and synthetic bile acids inhibition activity of LIFR/LIF binding accessed by a cell-free AlphaScreen assay, particularly in **(D)** LCA **(E)** CDCA and **(F)** BAR502 IC_50_ are shown. **(G)** STAT3 transactivation on HepG2 cells. The table **(H)** summarizes EC50 on FXR and GPBAR1 and IC_50_ on LIFR of Natural and synthetic bile acids.

Since there are robust evidence that the LIF/LIFR pathway exerts a pro-oncogenic role in PDAC cell lines (13, 20, 55), and because FXR expression is increased in human PDAC tissues, we have focused our attention on the role that natural and synthetic steroids exert in modulating pancreatic cancer cell lines. For these purposes, we have first carried out a series of docking calculations on hLIFR using a small library of natural steroids (**Figure 2**), including LCA and CDCA (56), and the semisynthetic bile alcohol steroidal agonist BAR502 (57).

The efficacy of LCA, CDCA and BAR502 as LIFR antagonists in a cell-free system was then measured using a well-consolidated platform based on Alpha Screen assay. The results of these studies reported in **Figure 2D** demonstrated that LCA and CDCA elicited a slight, thought significant, inhibitory effect on LIF/LIFR complex formation. In contrast, BAR502 could be considered as a potent LIFR antagonist that inhibits LIF/LIFR interaction with IC_50_ of 3.59 μM (**Figure 2F**), this result was confirmed by STAT3 transactivation assay performed in HepG2 cells, with an IC_50_ of 2.73 μM (**Figure 2G**).

Because BAR502 was significantly more potent than natural bile acids and is currently advanced into clinical trials (58), we have used this agent in the following experiments.

### BAR502 binds within loops 2 and 3 and disrupts LIF binding site

**Figure 3:**
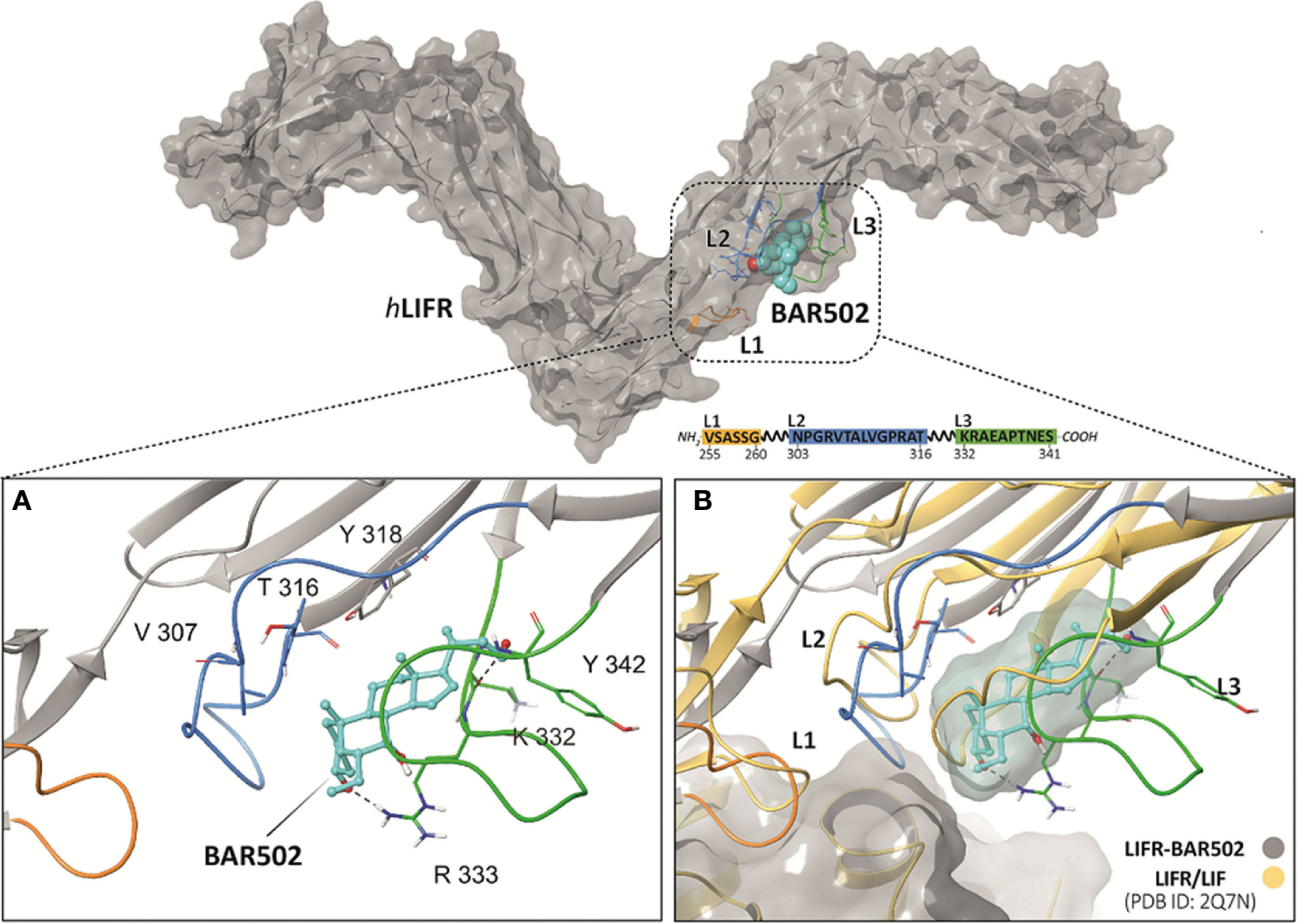
**(A)** View of the hLIFR-BAR502 representative cluster obtained after 100 ns of MD and **(B)** superimposed respect to the LIF-LIFR complex (PDB: 2Q7N). The pocket is defined by three loops, namely L1 (255-VSASSG-260), L2 (303-NPGRVTALVGPRAT-316), and L3 (332-KRAEAPTNES-341) (rectangular), which was already characterized as binding sites for EC359 and Mifepristone. L1, L2 and L3 are highlighted in yellow, blue, and green, respectively. BAR502 (cyan) and the relevant residues are labelled and coloured.

The binding between BAR502 and LIFR was investigated through a two-steps docking procedure followed by molecular dynamic (MD) simulation. Specifically, we have first used the QM-Polarized Ligand Docking (QPLD) protocol, whose best poses (-5.207 kcal/mol) were submitted to a second, more accurate Induced Fit Docking (IFD) analysis, that includes also the receptor flexibility. Given the high flexibility of the L1, L2 and L3 loops of the hLIFR binding site, the best pose obtained by IFD (-5.826 kcal/mol) was further refined using 100 ns of MD. From the analysis of the MD trajectory of BAR502, and of the ligand root means square deviation (L-RMSD) plot, it was found that after about 20 ns (**Supplementary Figure Sx1, A**), the ligand binding conformation was stabilized in a pocket defined by loops L2 and L3, with the 3-OH group anchored *via* H-bonding to the guanidine group of Arg333 (**Figure 3A**). The clustering analysis results showed that the MD trajectory of BAR502 produced two very similar binding conformations, accounting for 56% and 26% of the hypothetical binding poses, respectively (**Supplementary Figure Sx1B, C**). In both clusters, BAR502 bound to loops L2 and L3 (**Supplementary Figure SX2**), engaging hydrogen bond (H-bond) with the 3-OH to Arg333 and, discontinuously, to the backbone of Gly312. The 7-OH group established discontinuous H-bonds with the carbonyl backbone of Thr338. In the most populated cluster, the hydroxyl function at C23 H-bonds with Lys332, while in the second cluster, it was bound to the carbonyl group of the backbone of Tyr342. The B-C-D ring systems engaged hydrophobic interactions with residues from both L2 (Trp302, Val311 and Ala315), and L3 (Arg333 chain, Thr388 and Leu331) and the β-sheet (Tyr318). Moreover, the 6-ethyl group of BAR502 was firmly in contact with the Cβ of Asn339, helping to maintain the A and B rings in a “box” formed by Arg333, Asn339, Val311 and Ala315 (**Supporting Figure Sx2**). Overall, the MD of BAR502 highlights a significant alteration of the L2 and L3 loops conformation, thus causing the distortion of the LIF binding site (**Figure 3B**).

### LIFR antagonism exerts by BAR502 limits MIA PaCa-2 cells proliferation and migration

**Figure 4:**
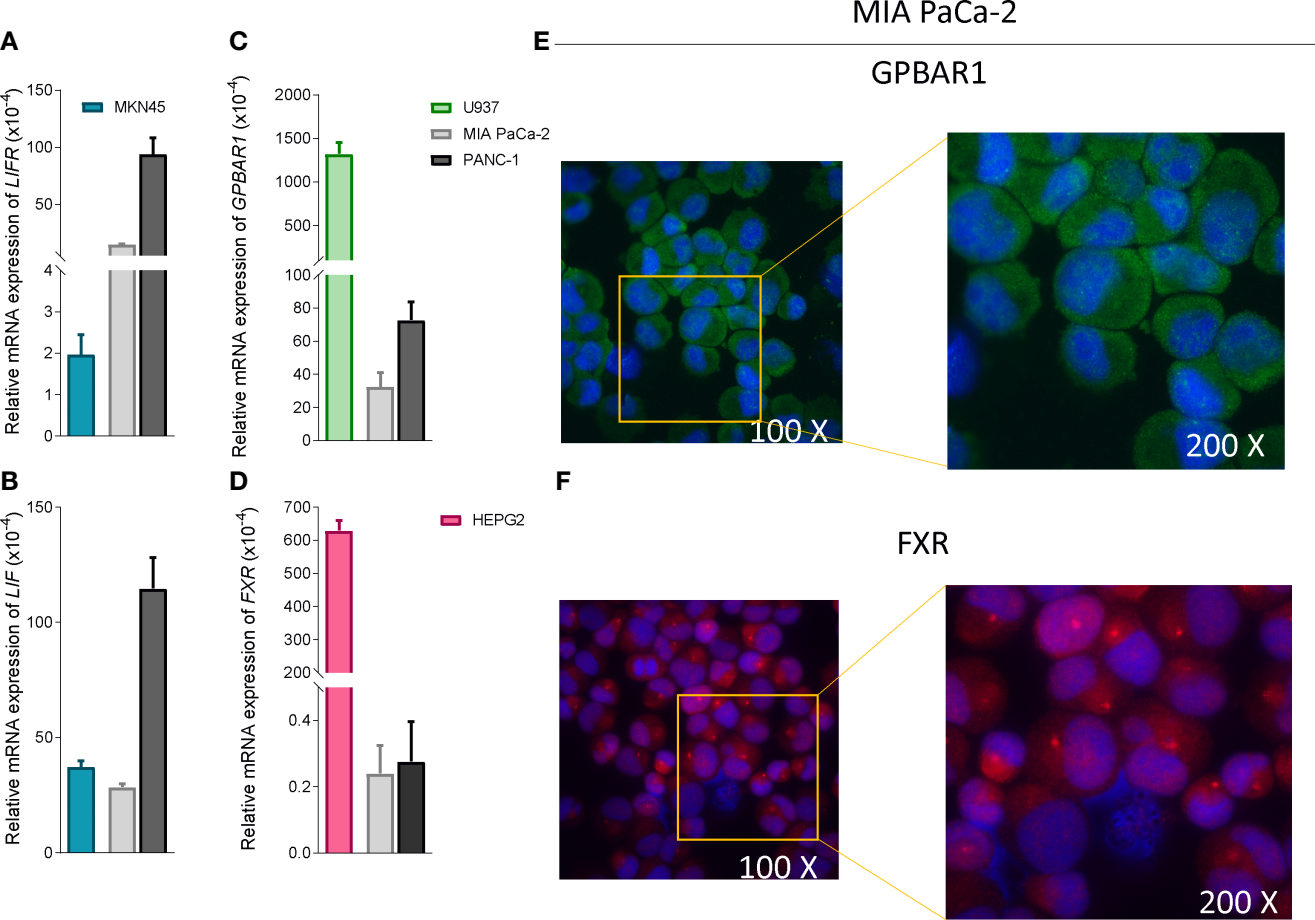
PDAC cells expressed low levels of bile acids receptors. Relative mRNA expression of **(A)** LIFR **(B)** LIF compared to MKN45 **(C)** GPBAR1 compared to U937 and **(D)** FXR compared to HEPG2. Each value is normalized to GAPDH and is expressed relative to those of positive controls, which are arbitrarily set to 1. Results are the mean ± SEM of three samples for group. Immunofluorescence analysis of **(E)** Gpbar1 and **(F)** Fxr in MIA PaCa-2 cells (Magnification 100x on left and 200x on right).

To functionally characterize the effect of BAR502 as LIFR antagonist, we have then performed *in vitro* assays using a human macrophage cell line, U937 cells, and liver, HEPG2, PDAC and gastric cancer, MKN45 cell lines (59). As shown in **Figures 4A, B**, PANC-1 exhibits highest levels of expression of LIF and LIFR compared to MKN45 cells. However, since our previous studies (20) have shown that MIA PaCA-2 cells are highly responsible to LIF, we have used this cell line for the following experiments.

In contrast to LIF/LIFR, the expression (mRNA and Immunofluorescence analysis) of GPBAR1 was almost undetectable in PDCA cell lines, as compared to U937 cells (**Figures 4C, E**), while PDAC cell lines express the nuclear receptor FXR (**Figures 4C, D, F**), though the expression was significantly lower than that detected in HEPG2 cells.

**Figure 5:**
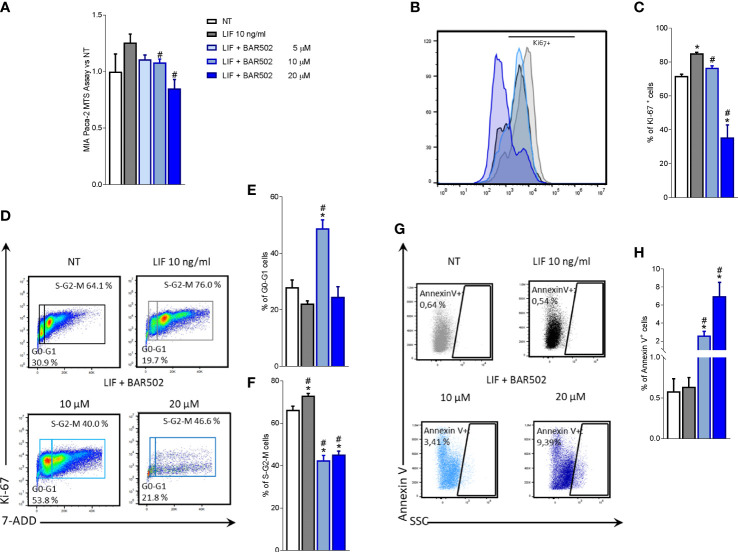
LIFR inhibition reverses PDAC cell proliferation promoted by LIF. MIA PaCa-2 cells were serum-starved and primed with LIF (10 ng/mL) alone or in combination with increasing concentrations of BAR502 (5, 10,20 μM). Data shown are **(A)** MTS assay performed on MIA PaCa-2. Each value is expressed relative to the non-treated (NT) value, which is arbitrarily set to 1. Results are the mean ± SEM of 10 samples per group. Cell cycle phase analysis was performed by Ki-67/7-AAD staining through IC-FACS. **(B)** Representative IC-FACS shows Ki-67 positive MIA PaCa-2 cells and **(C)** frequencies of Ki-67 positive cells. **(D)** Representative IC-FACS shows cell cycle fraction in each experimental group. Data shown are frequencies of cells in the **(E)** G0-G1 phase and **(F)** S-G2-M phase. **(G)** Representative IC-FACS shows Annexin V+ cells. **(H)** Data shown are frequencies of Annexin V+ single cells. Results are the mean ± SEM of five samples for group (* represents statistical significance versus NT, and # versus LIF, p < 0.05).

We have then investigated whether LIF acts as an autocrine factor to perpetuate PDAC cells growth and proliferation (60) and these effects were modulated by BAR502. For this purpose, MIA PaCa-2 cells, grown in a serum free medium, were exposed to 10 ng/mL LIF alone or in combination with increasing concentrations of BAR502 (5, 10, 20 µM) for 24 h. As shown in **Figure 5A**, BAR502 reversed the LIF-proliferative effect in a concentration-dependent manner.

The action of BAR502 on cell replication was also investigated by Ki-67/7-AAD IC-FACS staining (**Figures 5B-F**). More specifically, the analysis of Ki-67^+^ cells (**Figures 5B, C**) revealed that not only exposure to LIF increased the number of Ki-67 positive cells in a statistically-dependent manner, but shifted the fluorescence pick to the right, compared to cells left untreated (**Figure 5B**). This pattern was reversed by LIFR inhibition with BAR502 (**Figure 5B**). In addition, BAR502 (10-20 µM) modulates the cell cycle progression (**Figures 5C-F**) and the apoptosis cell rates, as assessed by Annexin V staining (**Figures 5G, H**). Together these results demonstrated that LIF increases the S-G2-M transition and that this effect was significantly reversed by BAR502 that also increased the percentage of Annexin V^+^ cells (p<0.05) (**Figure 5H**).

**Figure 6:**
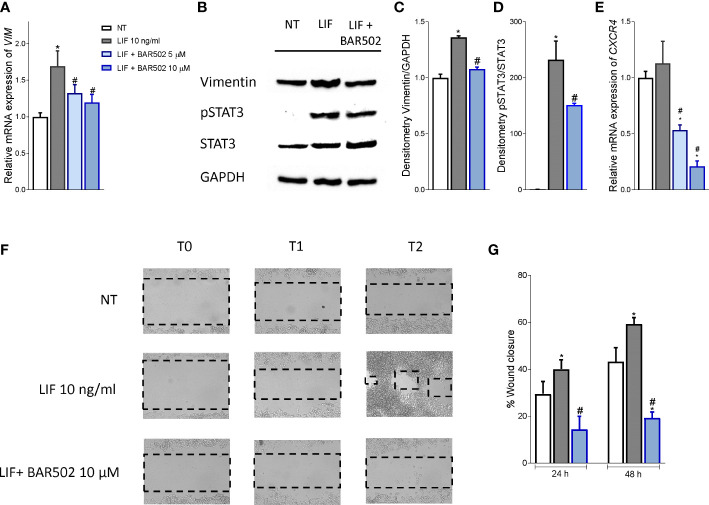
BAR502 inhibits *in vitro* migration in STAT3-dependent signalling. MIA PaCa-2 cells were serum-starved for 24 hours and exposed to LIF (10 ng/mL) alone or in combination with increasing concentrations of BAR502 (5, 10 μM) for 24 hours. Data displayed are: **(A)** Relative mRNA expression of the EMT markers, VIM. Each value is normalized to GAPDH and is expressed relative to those of positive controls, which are arbitrarily set to 1. **(B)** Representative Western blot analysis of Vimentin, phospho-STAT3 and STAT3 proteins in MIA-PaCa-2 cells exposed to LIF (10 ng/ml) alone or plus BAR502 (10 μM) for 20 minutes. **(C)** Densitometric analysis demonstrating Vimentin/GAPDH and **(D)** phospho-STAT3/STAT3 ratio. **(E)** Relative mRNA expression of the prognostic marker, CXCR4. Each value is normalized to GAPDH and is expressed relative to those of positive controls, which are arbitrarily set to 1. **(F, G)** Scratch wound healing assay. MIA PaCa-2 cell monolayers were scraped in a straight line using a p200 pipette tip; then, they were left untreated or primed with LIF 10 ng/mL alone or in combination with BAR502 10 µM. The wound generated was captured at 0, 24 and 48 h of incubation with the compounds above described. The images show cell migration at the three times point indicated. **(G)** Images of obtained points were analysed, measuring scraped area and its closure *vs* the first time point at 0 (h) Results are the mean ± SEM of five samples per group (* represents statistical significance versus NT, and # versus LIF, p < 0.05).

Since the LIF/LIFR axis promotes EMT in various cell systems (13), we have then investigated whether BAR502 also reverses EMT features in MIA PaCa-2 cells and found that BAR502 (10-20 μM) reversed the induction of vimentin expression, RNA (**Figure 6A**) and protein (**Figures 6B, C**) caused by LIF. Furthermore, BAR502 significantly attenuated STAT3 phosphorylation caused by LIF (**Figures 6B-D**). The inhibition of LIFR exerted by BAR502 also reversed the mRNA expression of the pro-inflammatory factor CXCR4, whose expression was increased by LIF (**Figure 6E**). Since CXCR4 overexpression is a strong prognostic marker of lymph node involvement and metastasis development in PDAC, this finding might have a translational readout (61).

Since the above mentioned data demonstrated that BAR502 prevents the acquisition of a migratory phenotype, we have measured the motility of MIA PaCA-2 cells using a wound healing assay (**Figure 6F**). To this end, MIA PaCa-2 cells were growth in a complete serum starved DMEM medium and after the production of a scratch (Day 0), cells were exposed to 10 ng/mL LIF, alone or in combination with BAR502 (10 µM). The gain in the capacity of cells to differentiate into a migratory phenotype was calculated as the area between the two scratch edges at prespecified time points: 0 h, 24h and 48 h. As illustrated in **Figures 6F, G**, LIF induced cell migration and promoted the wound closure with a reduction of the scratch area by ≈ 16%. These findings were reversed by treatment with BAR502 that significantly decreased MIA PaCa-2 detachment and migration, with a reduction of ≈40% compared to LIF (p<0.05) (**Figure 6G**).

Altogether these findings suggest that LIFR antagonism in PDAC cell lines reduced cell proliferation and migration by reducing STAT3 phosphorylation.

### BAR502 anti-cancer activity is due to LIFR inhibition

**Figure 7:**
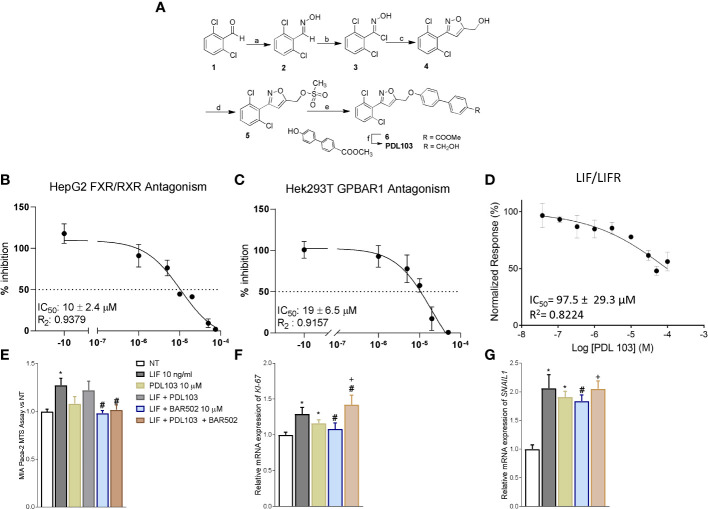
The effect of BAR502 is mediated through selectively LIFR inhibition. **(A)** Scheme 1. Reagents and conditions: a) NH_2_OH·HCl, NaOH, EtOH, Δ, 98%; b) N-clorosuccinimide, dry DMF, 0°C, 95%; c) propargylic alcohol, NaHCO_3_, CuSO_4_·5H_2_O, sodium ascorbate, t-BuOH/H_2_O 1:1, quantitative yield; d) methanesulfonyl chloride, TEA, dry THF, 92%; e) methyl 4’-hydroxy-4-biphenylcarboxylate, K_2_CO_3_, dry DMF, 0°C, 66%; f) LiBH_4_, MeOH, dry THF, 0°C, 82%. The synthetic strategy was as following: the commercially available 2,6-dichlorobenzaldehyde (1) was treated with hydroxylamine hydrochloride to form the oxime (2) which was in turn chlorinated with NCS to afford the chloro oxime (3). The 3,5-disubstituted isoxazole 4 was easily obtained as only regioisomer *via* [3 *+* 2]-cycloaddition between the 2,6-dichloro-N-hydroxybenzimidoyl chloride (3) and propargyl alcohol in presence of NaHCO_3_, catalytic CuSO_4_·5H_2_O and sodium ascorbate with quantitative yield. The intermediate 4 was then reacted with mesyl chloride and triethylamine to afford the mesyl ester (5) which was in turn coupled to methyl 4’-hydroxy-4-biphenylcarboxylate to afford the methyl esters 6. Finally, reduction with LiBH_4_ gave PDL103. **(B, C)** Antagonistic effects of PDL103 on FXR and GPBAR1 transactivation induced by CDCA and LCA, respectively, in HepG2 cells. **(D)** PDL103 inhibition activity of LIFR/LIF binding accessed by a cell-free AlphaScreen assay. **(E)** MTS assay performed on MIA PaCa-2. Each value is expressed relative to the non-treated (NT) value, which is arbitrarily set to 1. Results are the mean ± SEM of 10 samples per group. Relative mRNA expression of the proliferative marker (* represents statistical significance versus NT; # versus LIF; + versus PDL103) **(F)** KI-67 and **(G)** the EMT marker SNAIL1. Each value is normalized to GAPDH and is expressed relative to those of positive controls, which are arbitrarily set to 1. Results are the mean ± SEM of 5 samples per group.

To tight the biological effect of BAR502 to the LIF/LIFR antagonism, we have synthesized a dual FXR and GPBAR1 antagonist (**Figure 7A**). To this end we have generated a small library of 3,5-disubstituted isoxazole derivatives as potential dual FXR and GPBAR1 antagonists and tested them in a transactivation assay on FXR and GPBAR1 (data not published). From this library, PDL103 was proven to be a relatively potent novel dual FXR/GPBAR1 antagonist (IC_50_ = 10 µM and 19 µM, respectively) (**Figures 7B, C**). PDL103 was also tested in Alpha screen on LIF/LIFR. However, the result shown in **Figure 7D**, demonstrated that this compound was inactive towards LIF/LIFR complex. Because PDL103 is a dual FXR and GPBAR1 antagonist but is neutral toward LIF/LIFR, this agent represents an useful tool to rule out the involvement of the two receptors in the observed antagonism exerted by BAR502 on the LIF pathway. Indeed, as shown in **Figures 7E-G**, co-treating MIA PaCa-2 cells with this agent failed to reverse the effect of BAR502 on LIF/LIFR induced proliferation (Panel E and F) and EMT (Panel G).

### RNAseq analysis of the effects of BAR502 on MIA PaCa-2 cells

**Figure 8:**
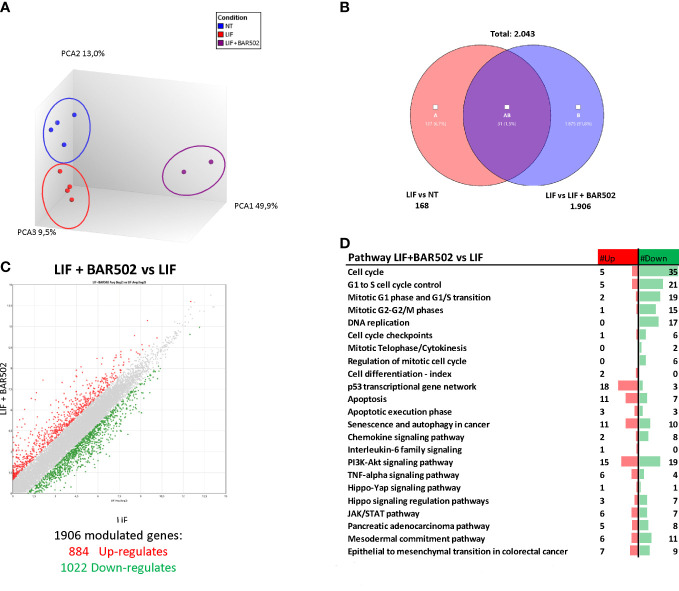
RNA-seq analysis of BAR502 effects on MIA PaCa-2 cells exposed to LIF alone or in combination with BAR502. MIA PaCa-2 cells were serum-starved for 24 hours and exposed to LIF (10 ng/mL) alone or in combination with increasing concentrations of BAR502 (10 μM) for 24 hours. **(A)** Heterogeneity characterization of the three experimental groups as shown by principal component analysis (PCA) plot. **(B)** Venn diagram of differentially expressed genes showing the overlapping region between the three experimental groups. **(C)** Scatter plots of transcripts differentially expressed between different experimental groups (fold change <−2 or >+2, p value < 0.05). Red dots represent significantly upregulated genes, and green dots represent significantly downregulated genes. **(D)** Table showing pathway modulated by LIF plus BAR502 versus LIF.

To further characterize the transcriptional profile modulated by exposure to LIF and BAR502, a AmpliSeq Transcriptome analysis (RNAseq) was conducted on MIA PaCa-2 cells left untreated or challenged with LIF alone or in combination with BAR502 (10 µM). The Principal Component Analysis (PCA) of the resulting transcriptome (**Figure 8A**) highlighted major dissimilarities between MIA PaCa-2 left untreated or treated with LIF and LIF/BAR502. **Figure 8B** displayed the Venn Diagram analysis of differentially expressed transcripts. As shown in **Figure 8B**, the analysis identified 2.043 transcripts differentially regulated across the three experimental groups: 168 transcripts were differentially modulated by LIF *versus* control cells (Subset A); 1.906 transcripts were differentially modulated by exposure to LIF/BAR502 in comparison to LIF alone (Subset B), while the AB subset includes only 31 transcripts that were modulated by LIF and LIF/BAR502 in comparison to control (NT) cells. The Scatter Plot (**Figure 8C**) of the 1.906 transcripts demonstrated that 884 transcripts were up-regulated and 1022 were down-regulated (**Figure 8C**). Then, the *per pathways* analysis of these differentially expressed transcript sets was performed using the TAC software (Affymetrix) to inspect the molecular pathways modulated by the exposure of MA PaCa-2 cells to LIF and BAR502. As illustrated in **Figure 8D**, the higher number of downregulated genes belong to the cell cycle (35 genes), G1 to S cell cycle control (21 genes), mitotic G1 phase and G1/S transition (19 genes), mitotic S-G2/M phases (15), DNA replication (17 genes), PI3K-Akt signalling pathway (19 genes). In contrast, the highest up-regulated genes fell in to the p53 transcriptional gene network (18 genes) and Apoptosis (11 genes) (**Figure 8D**). Within the genes that belonged to these pathways, the most downregulated gene by BAR502 was the *Kinesin Family Member 20A* (KIF20A) with a Fold Change (FC) of -17,49. KIF20A is a motor kinesin protein involved in mitosis process (62). The overexpression of KIF20A occurs in several tumours, including gastric cancer (GC) (63), lung cancer (64), cervical cancer (65), glioma (66) and also PDAC (67). In addition to KIF20A, BAR502 potently downregulated the expression of the *Ribonucleotide reductase subunit M2* (RRM2), (FC: -14,34) (68) and *TNF Receptor Superfamily Member 1B* (TNFRSF1B or TNFR2) (FC: -11,72) (69) and *DNA topoisomerase II alpha* (TOP2A) (FC: -9,61), an important regulator of DNA replication and cell cycle progression and up-regulated in PDAC (70). On the other hand, exposure to BAR502 increased the expression of a number of genes, including the *Solute carrier family 7 member 11* (SLC7A11) (FC: 35,54), a cysteine transporter involved in the inhibition of the ferroptosis programmed cell death (71), that was the most upregulated gene, and the *Cyclin D2* (CCND2) (FC: 16,13) (72) and *Sestrin 2* (SESN2) (FC: 15,84) (73),, whose expression are robustly reduced in PDAC cells (74).

## Discussion

LIF is the most pleiotropic member of the IL-6 family of cytokines and controls multiple biological functions, including the stem cell ability to “self-renew”, the embryonic implantation and placental formation and cell proliferation and differentiation (10). LIF exploits its function by binding to an heterodimeric membrane receptor complex assembled by the LIFR and glycoprotein 130 (gp130) (12). LIFR lacks an intrinsic tyrosine kinase activity, but either LIFR and gp130 are constitutively associated with of cytoplasmic tyrosine kinases belonging to the Jak family (55). Consequently, binding of LIF to LIFR induces the assembly of the heterodimeric complex LIFR:gp130 and promotes a Jak-Tyk phosphorylation and propagation of downstream signalling (75).

The LIF/LIFR axis plays a central role in tumour growth and progression, regulating key aspects of cancer biology including cancer cell growth, proliferation, migration and chemotherapy resistance (76). Consistent with this view, an aberrant production of LIF and/or an increase in the circulating levels of LIF correlate with tumour chemoresistance in several solid cancers (60).

LIF acts as a growth factor in PDAC cells (12) and high levels of LIF expression occur in human PDAC and correlate with a shorter overall survival (12). LIF/LIFR signalling promotes tumorigenesis and metastasis by the upregulation of LIF/LIFR-JAK-STAT3 signalling *via* autocrine and paracrine mechanisms (77). We have previously demonstrated that inhibition of the LIF/LIFR axis reversed the increased proliferation rate and propensity to develop a EMT phenotype in MIA PaCa-2 and PANC-1 cells. More specifically we have reported that the small steroidal molecule LIFR inhibitor, EC359, reduced the mRNA expression of VIM and Snail1, validating the potential role of LIFR as therapeutic target in PDAC (20).

Prompted by these findings and by the fact that steroids such as mifepristone, an antiprogesterone agent, effectively counteracted the effects of LIF on PDAC cells, we have embarked in a screening project of an in-house library of natural and synthetic bile acids. This screening allowed us to show that LCA, CDCA and BAR502 exert LIFR antagonism. By Alpha screen assay, we have then confirmed that BAR502 is a potent LIFR inhibitor with an IC_50_ of 3.59 µM. Traditionally, bile acids have been linked to development of gastrointestinal and liver cancers, but the putative mechanisms have remained elusive. In contrast, a number of recent investigations, as detailed in the introduction, have shown the opposite, suggesting that bile acids might exert anti-tumour effects in solid cancers (26), but these effects are strictly dependent on their concentrations, cellular microenvironment and expression of key receptors such as FXR and GPBAR1 (30). In general, it appears that low concentrations of bile acids exert anti-cancer effects, while in super-physiological concentrations, bile acids promote cell proliferation, migration and invasion. This phenomenon is due to their amphipathic structure and the activation of off-target mechanisms not observed at physiological concentrations.

By computational analysis we have clarifies the structural requirement for the binding to LIFR. Our results indicated that natural bile acids and BAR502 bind to the same pocket within loops L2 and L3 of LIFR. Because these two loops are involved in LIF binding to hLIFR, we speculated that antagonism of BAR502 against LIFR is due to the ability of this agent to prevent LIF/LIFR binding. Moreover, molecular dynamic analysis of the BAR502 in conjunction with LIFR showed a stable binding mode of BAR502 over the time of the simulation. The binding was stabilized by H-bonds of the ligand 3-, 7- and 23-OH, and by hydrophobic contacts with both L2, L3 and β-sheet residues with the steroidal agent. Importantly we found that the 6-ethyl group contributed to further stabilize the binding mode through the contact with the Cβ of Asn339, entrapping the A and B rings in a box formed by Arg333, Asn339, Val311 and Ala315 (**Figure 3A**). The computational results highlighted that the binding of BAR502 within loops L2 and L3 might impact with the position of L2 and L3 widening the distance between the two loops (**Figure 3B**), likely affecting the 3D structure of the whole LIF binding site.

To further characterize functionally the relevance of LIFR inhibition caused by BAR502, we have assessed whether BAR502 counteracts the effects exerted by LIF in MIA PaCa-2 cells. The results of these experiments were consistent with the cell-free assay and demonstrated that BAR502 effectively counteracted the pro-oncogenic effects caused by LIF in a concentration-dependent manner and in a FXR/GPBAR1 independent manner, reducing cell vitality, the number ki-67+ cells and increasing the frequencies of cells in the resting G0-G1 cell cycle phase, blocking S-G2-M transition and increasing the frequencies of AnnexinV^+^ apoptotic cells. Similarly, BAR502 reversed EMT features, diminished the regulation of Vimentin, CXCR4 and the gain of the migratory phenotype, and STAT3 phosphorylation induced by LIF, further suggesting a potential utility in counteracting the pro-oncogenic activity of LIF/LIFR pathway.

In order to better dissect the molecular mechanisms that mediates anti LIF/LIFR effects of BAR502, we have carried out a RNAseq analysis on MIA PaCa-2 cells exposed to LIF. The results of these studies demonstrated that antagonism of LIF/LIFR exerted by BAR502 was supported by regulation of the expression of large group of genes, including 35 genes involved in the Cell cycle modulation, 21 genes involved in G1 to S cell cycle control, 19 genes in G1-S phase transition, 17 genes involved in DNA replication, 15 genes involved in the G2/M shift, 18 in p53 transcriptional gene network and 11 in apoptosis. The most downregulated of these genes was KIF20A, a motor kinesin protein involved in mitosis process and in the trafficking of organelles and vesicles. Positive expression of KIF20A correlates with a poor prognosis and tumour growth and progression in early-stage of several types of cancer including breast (78), colorectal (79) and cervical cancers (65) but also PDAC (67) and glioma (80). Overexpression of KIF20A enhances resistance to chemotherapy (79) while KIF20A inhibition reduces cell proliferation, migration and invasion of pancreatic cancer cells in PDAC (67). In addition to KIF20A, BAR502 reduced the expression of LIF-induced RRM2 in MIA PaCA-2 cells. Expression RRM2 correlates with a poor prognosis in several tumours including lung cancer (81) and PDAC (82). Also, RRM2 is a validated biomarker of sensitivity of PDAC to chemotherapy, and it is demonstrated that the high levels of RRM2 predict poor prognosis and resistance to gemcitabine in PDAC patients (66, 83).

Another gene that was downregulated by BAR502 in LIF-challenged MIA PaCa-2 cells was TNFR2, one of two membrane receptors that binds tumour necrosis factor-alpha (TNFα) (84). TNFR2 is expressed by immunomodulatory cells such as myeloid-derived suppressor cells (MDSCs) (85) and regulatory T cells (Tregs) (86), and plays a central role in their homeostasis by regulating their expansion, enhancing their phenotypic stability and immune-suppressive abilities (87). High expression of TNFR2 is a characteristic of tumour-associated Treg that promotes cancer growth by hindering the anti-tumour immune responses (85, 88, 89). TNFR2 regulates the transcription of PDL1 *via* the p65 NF-κB pathway, suggesting that BAR502 by downregulating the expression of TNFR2, might restore immune surveillance of pancreatic cancer cells in PDAC (69).

**Figure 9:**
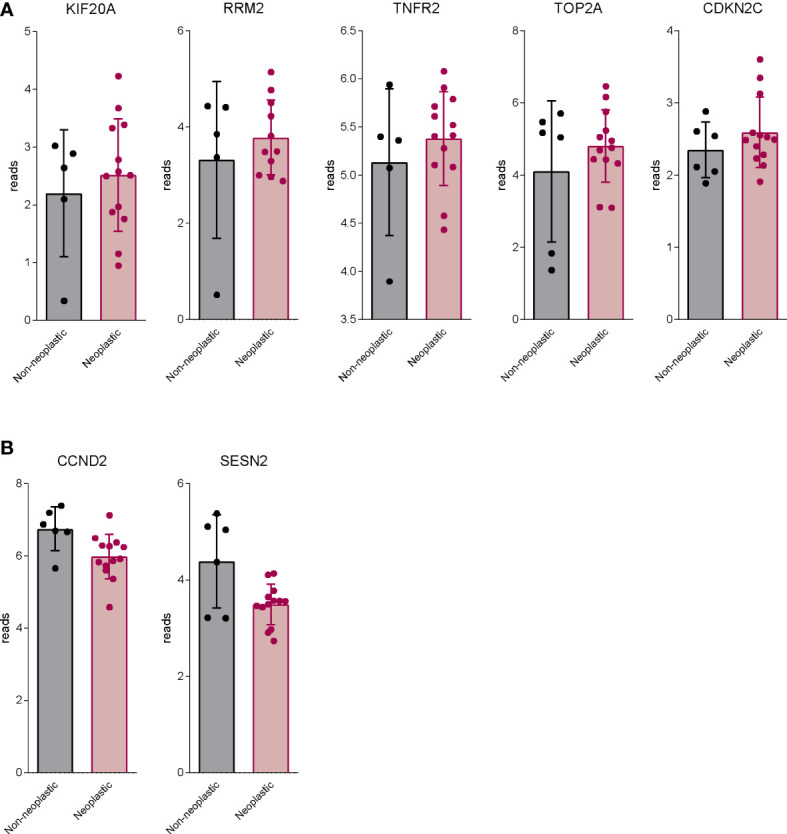
BAR502 modulated genes generally regulated aberrantly in PDAC. RNA-seq analysis of non-neoplastic and neoplastic mucosa of PDAC from GSE196009 repository. Each dot represents a patient. Data shown represent the gene profile expression of **(A)** genes upregulated in human PDAC, which are downregulated by BAR502 exposure in MIA PaCa-2 cells. **(B)** genes down-regulated in human PDAC, which are upregulated by BAR502 exposure in MIA PaCa-2 cells.

BAR502 also modulated the expression of a groups of genes whose expression is usually suppressed in neoplastic tissues in comparison to non-neoplastic counterparts (**Figure 9**). Exposure to BAR502 robustly increased the expression of SSN2, a highly conserved stress-induced protein. SSN2 is secreted by macrophages, T lymphocytes and epithelial cells, in a wide variety of stress conditions such as oxidative stress, hypoxia or DNA damage, and inhibits the accumulation of reactive oxygen species (ROS) through the activation of the nuclear factor-erythrocyte 2-related factor (Nrf2) signalling. SSN2 plays a tumour suppressive role by the inhibition of tumour growth and the activation of autophagy process, regulating the mTOR/AMPK signalling pathway (90).

In summary, by molecular modelling and pharmacological experiments, we have shown that BAR502 binds LIFR and acts as LIF/LIFR inhibitor. BAR502, a semisynthetic bile alcohol steroidal agonist (42), functions as a potent LIFR antagonist, directly binding within the loops L2 and L3 of the Ig-like domain of LIFR, and preventing its activation and signalling. BAR502 decreases PDAC cell proliferation and slows down cell cycle progression, arresting PDAC cells in the G0–G1 phases and retarding the transition toward S-G2-M phase. BAR502 promotes the apoptosis of PDCA cells and reverses the migratory phenotype induced by LIF.

The present study has several limitations. The most relevant of which is that the role of LIF/LIFR system has been tested in *in vitro* models and therefore the real anti-cancer potential of BAR502 in PDAC should be further investigated in clinically relevant settings.

In conclusion, in the present study we have described a dual GPBAR1/FXR agonist as a potential antagonist of LIFR and suggested that BAR502 could be used to regulate the LIF/LIFR pathway in relevant clinical settings such as LIF overexpressing-PDAC.

## Data availability statement

The datasets presented in this study can be found in online repositories. The names of the repository/repositories and accession number(s) can be found in the article/**Supplementary Material**.

## Author contributions

SF, AZ and BC contributed to conception and design of the study. AZ and SF provided research funding. CG, SM, MBi, AL, FM, MM and MBo performed the data analysis. CG, MBi, AL, FM, MM, VS, MBo and PRa performed the statistical analysis. SF, CG, AL, BC, VS, EM, PRi and ED wrote the manuscript. CG, SM, RR, MBo, RB, CM, GU, RR, AL, EM and MM contributed to the experiments. All authors contributed to manuscript revision and read and approved the submitted version. All authors contributed to the article and approved the submitted version.

## Supplementary material

Supplementary Figure 1
**(A)** The ligand root means square deviation (L-RMSD) plot; **(B)** cluster analysis and **(C)** cluster distribution plot of hLIFR-BAR502 complex after 100ns of MD simulation.

Supplementary Figure 2 Different views (frontal, 35° and 90°) of the two representative clusters (cl0 and cl1) of the hLIFR-BAR502 complex after 100ns of MDs. L1, L2 and L3 are highlighted in yellow, blue, and green, respectively. BAR502 and the relevant residues are labelled and coloured.

Supplementary Figure 3 Table showing genes modulated by LIF in combination with BAR502 versus LIF resulted by RNA-seq analysis.

Supplementary Figure 4 MIA PaCa-2 cells were serum starved and exposed to vehicle or LIF (10 ng/ml) alone or in combination with BAR502 (10 µM) for 24 h. The map shows the pathway main regulated by BAR502 administration. The upregulated genes (Fold Change < −2 or > 2, p value < 0.05) are represented in the map in red and the downregulated genes are in green.
