# Supplementary figures and images for: Discovery of BAR502, as potent steroidal antagonist of leukemia inhibitory factor receptor for the treatment of pancreatic adenocarcinoma
Source: Front Oncol. 2023 Mar 14;13:1140730. doi: 10.3389/fonc.2023.1140730 (PMC10043345; doi:10.3389/fonc.2023.1140730)

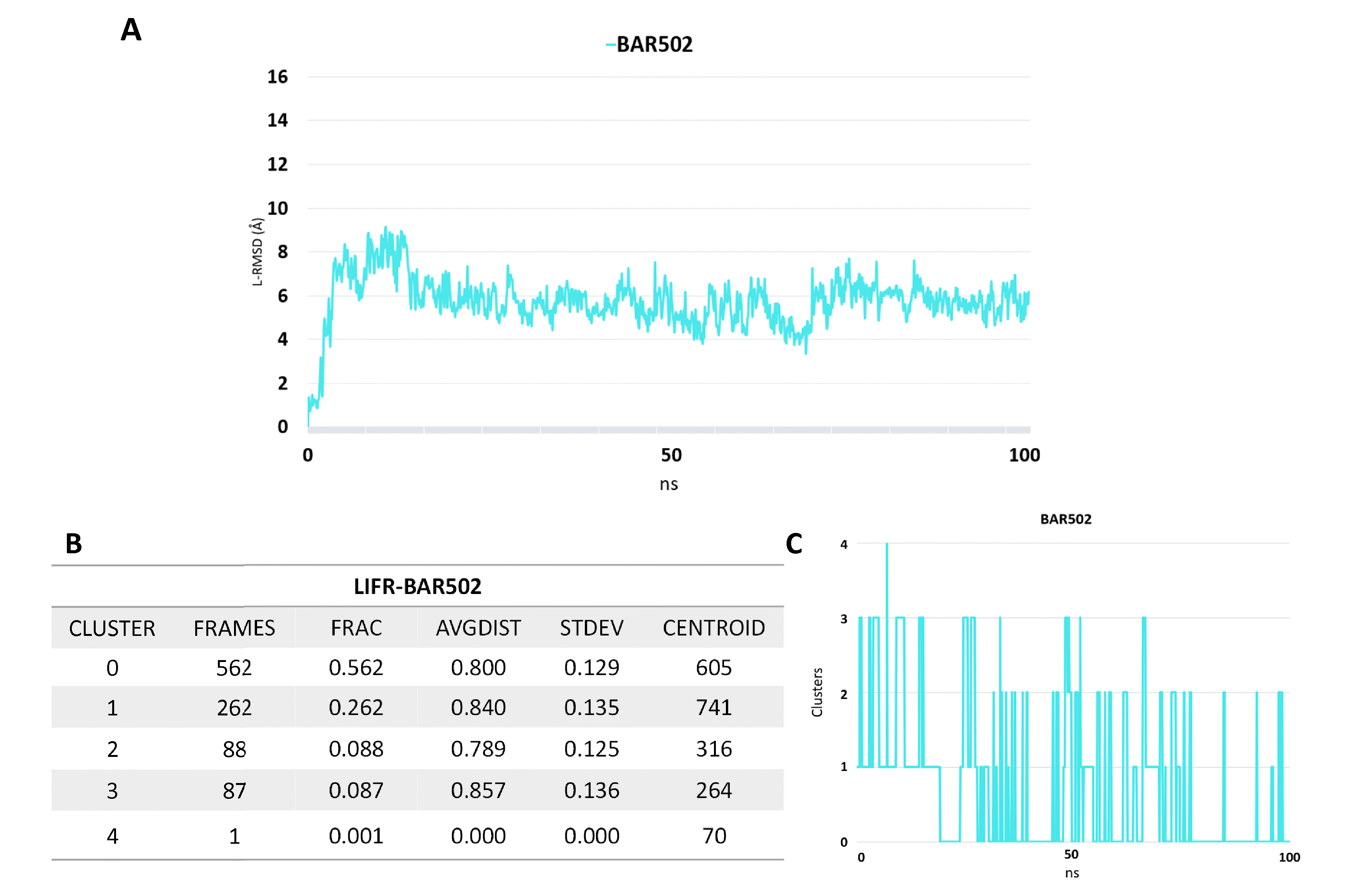

Supplement: Supplementary Figure 1 — (A) The ligand root means square deviation (L-RMSD) plot; (B) cluster analysis and (C) cluster distribution plot of hLIFR-BAR502 complex after 100ns of MD simulation. [file Image_1.tif]

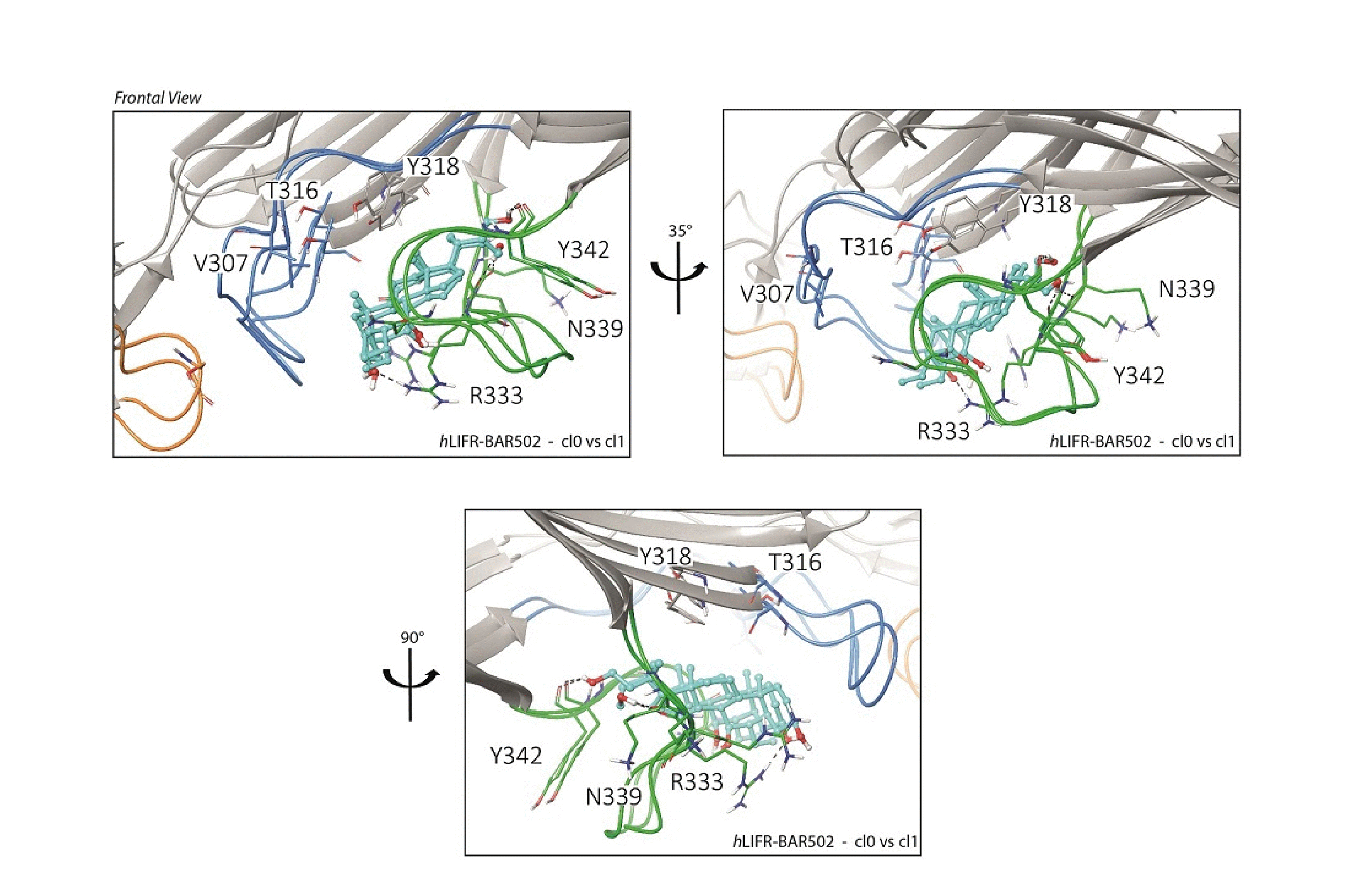

Supplement: Supplementary Figure 2 — Different views (frontal, 35° and 90°) of the two representative clusters (cl0 and cl1) of the hLIFR-BAR502 complex after 100ns of MDs. L1, L2 and L3 are highlighted in yellow, blue, and green, respectively. BAR502 and the relevant residues are labelled and coloured. [file Image_2.tif]

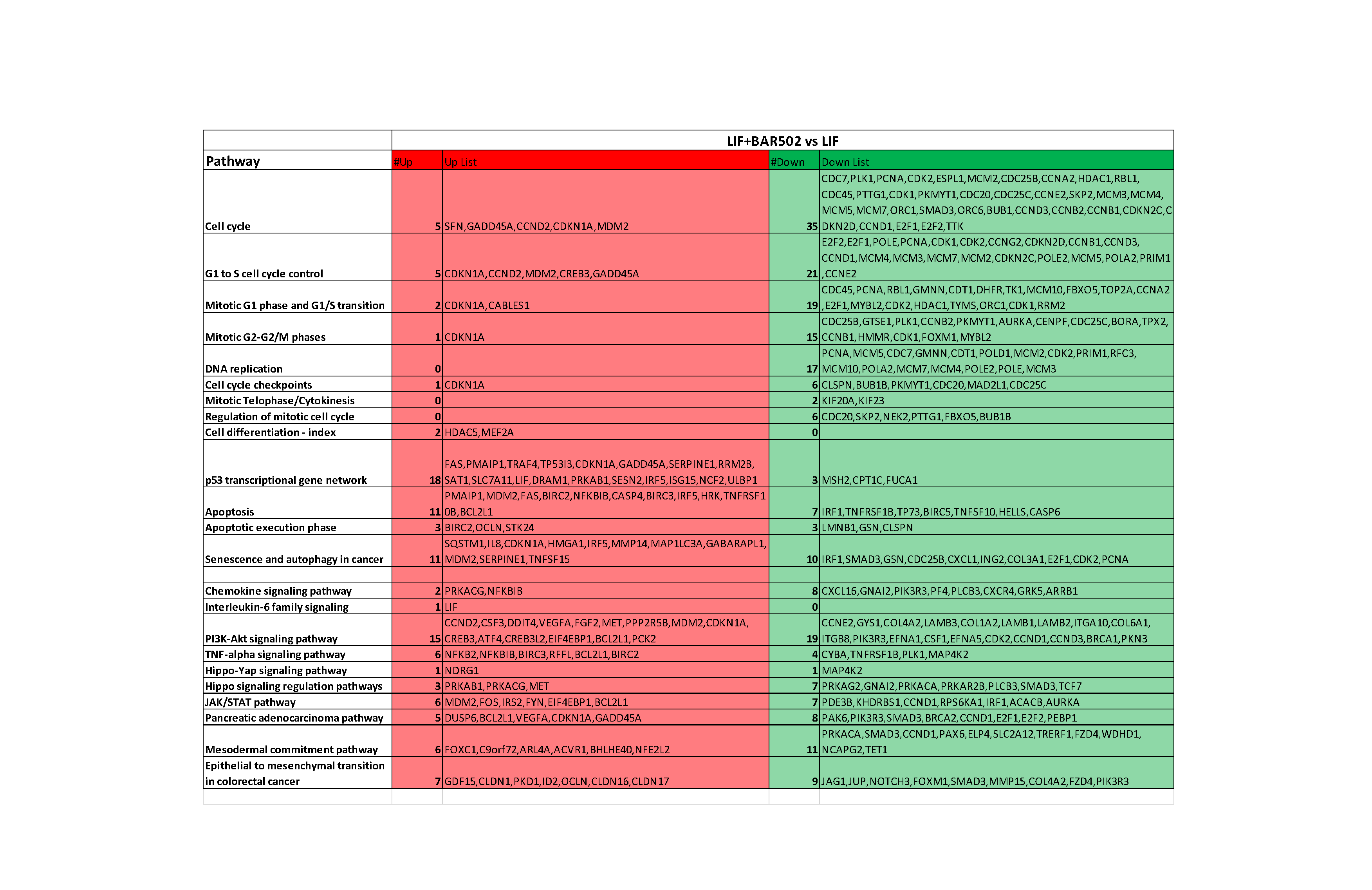

Supplement: Supplementary Figure 3 — Table showing genes modulated by LIF in combination with BAR502 versus LIF resulted by RNA-seq analysis. [file Image_3.tif]

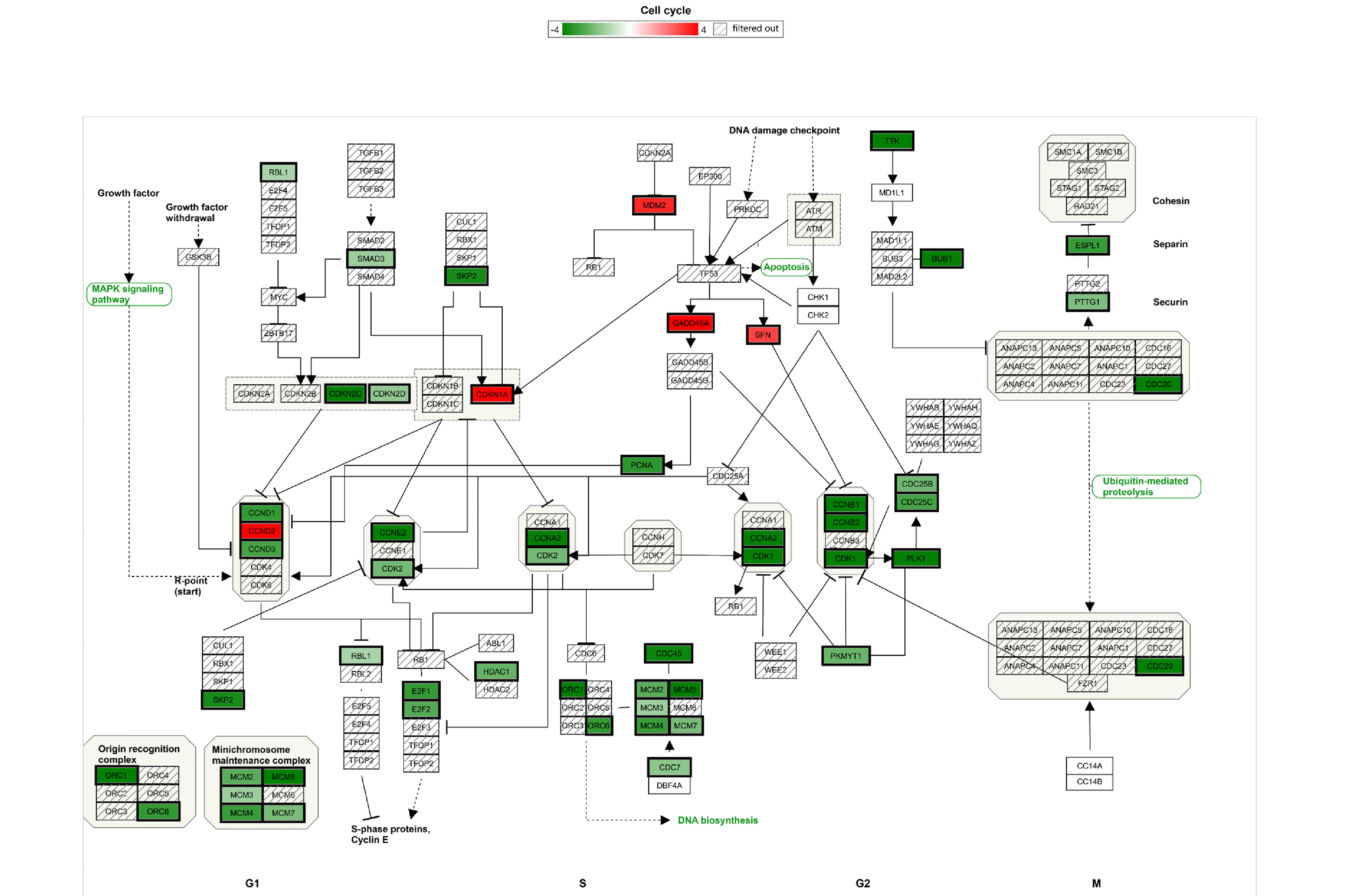

Supplement: Supplementary Figure 4 — MIA PaCa-2 cells were serum starved and exposed to vehicle or LIF (10 ng/ml) alone or in combination with BAR502 (10 µM) for 24 h. The map shows the pathway main regulated by BAR502 administration. The upregulated genes (Fold Change < −2 or > 2, p value < 0.05) are represented in the map in red and the downregulated genes are in green. [file Image_4.tif]
